# Large-scale determinants of street tree growth rates across an urban environment

**DOI:** 10.1371/journal.pone.0304447

**Published:** 2024-07-11

**Authors:** Brian J. Mailloux, Clare McGillis, Terryanne Maenza-Gmelch, Patricia J. Culligan, Mike Z. He, Gabriella Kaspi, Madeline Miley, Ella Komita-Moussa, Tiffany R. Sanchez, Ella Steiger, Haokai Zhao, Elizabeth M. Cook

**Affiliations:** 1 Environmental Science Department, Barnard College, New York, NY, United States of America; 2 Department of Civil Engineering & Engineering Mechanics, Columbia University, New York, NY, United States of America; 3 Department of Civil and Environmental Engineering and Earth Sciences, University of Notre Dame, Notre Dame, Indiana, United States of America; 4 Department of Environmental Medicine and Public Health, Icahn School of Medicine at Mount Sinai, New York, NY, United States of America; 5 Department of Environment Health Sciences, Mailman School of Public Health, Columbia University, New York, NY, United States of America; New York University Grossman School of Medicine, UNITED STATES

## Abstract

Urban street trees offer cities critical environmental and social benefits. In New York City (NYC), a decadal census of every street tree is conducted to help understand and manage the urban forest. However, it has previously been impossible to analyze growth of an individual tree because of uncertainty in tree location. This study overcomes this limitation using a three-step alignment process for identifying individual trees with ZIP Codes, address, and species instead of map coordinates. We estimated individual growth rates for 126,362 street trees (59 species and 19% of 2015 trees) using the difference between diameter at breast height (DBH) from the 2005 and 2015 tree censuses. The tree identification method was verified by locating and measuring the DBH of select trees and measuring a set of trees annually for over 5 years. We examined determinants of tree growth rates and explored their spatial distribution. In our newly created NYC tree growth database, fourteen species have over 1000 unique trees. The three most abundant tree species vary in growth rates; London Planetree (n = 32,056, 0.163 in/yr) grew the slowest compared to Honeylocust (n = 15,967, 0.356 in/yr), and Callery Pear (n = 15,902, 0.334 in/yr). Overall, Silver Linden was the fastest growing species (n = 1,149, 0.510 in/yr). Ordinary least squares regression that incorporated biological factors including size and the local urban form indicated that species was the major factor controlling growth rates, and tree stewardship had only a small effect. Furthermore, tree measurements by volunteer community scientists were as accurate as those made by NYC staff. Examining city wide patterns of tree growth indicates that areas with a higher Social Vulnerability Index have higher than expected growth rates. Continued efforts in street tree planting should utilize known growth rates while incorporating community voices to better provide long-term ecosystem services across NYC.

## Introduction

Urban streets trees provide many benefits or ecosystem services [2–5]. Cities, including New York City (NYC), Los Angeles, and Chicago, are undertaking programs to increase the number of street trees in an effort to increase ecosystem services, or the contributions of nature to people, and ensure their equitable distribution [6–9]. Urban trees are often planted as small saplings. Many residents prefer the benefits of larger street trees, but larger trees also pose challenges with pruning needs and possible disservices, such as pollen and pavement damage, but may also influence crime [10–17]. However, it is only after decades of growth that trees mature and many of the ecosystem services provided by urban trees are realized [18–22]. In order to make planning decisions [23], minimize environmental injustices [24–26], and predict future ecosystem services [27], it is important to understand the effect of biological properties and urban form on street tree growth rates.

Studies examining urban tree growth have traditionally focused on forest patches [28], examined regional variations [29] or used urban-to-rural gradients to simulate the impacts of future climate change [e.g. 30, 31]. Urban-to-rural gradients have highlighted the effect of multiple parameters on growth rates [32–34]. For example, in studies across the Eastern United States, transitioning from rural forests to urban forests showed more rapid growth and basal area increments in the urban setting than a rural setting [33, 35, 36]. Similarly in Berlin, dense urban environments with higher temperatures and less precipitation had higher growth rates [37]. Yet, other research examining multiple species across cities in the United Kingdom had varied results [38].

However, street trees are different than urban forest patches. In addition to regional climatic characteristics related to temperature and precipitation, urban form (local built urban environment) and social factors, including local stewardship, impact urban street tree growth rates [39–45]. Street trees—located on or near a road—are often planted in tree pits (small cutouts in sidewalks), or in elongated areas of soil between roads and/or a sidewalk (often termed a road verge or tree lawn). In Central Ohio, urban trees within 2m of pavement had slower growth rates compared to nearby woodlots [32]. City inspectors in Montreal tracked the long-term average growth rate of street trees and found that tree species, side of the street (north, south, east, or west), obstructions such as signage, and zoning all had an impact on growth rates [46]. Other studies have focused on tree pits and found varied results suggesting size, soil type, and design may impact tree growth [47, 48]. In addition, social factors and management, including stewardship, tree planting by larger, well established groups, and socio-demographic characteristics have been shown to positively impact growth [49]. As trees grow and mature, the costs and benefits change; thus accurate growth estimates could aid urban planning as municipalities try to maximize ecosystem services. However, few citywide studies or databases of street tree growth are available.

Monitoring street tree growth rates across an entire city—regardless of city size—is time consuming and requires substantial effort. Tree growth rates can be determined by measuring annual tree rings obtained from tree cores, measuring the tree’s diameter at breast height (DBH) and knowing the age of a tree, or through repeated measurements of DBH. Measuring cores can give detailed annual data over long-time spans but is labor intensive and requires specialized equipment. Instead, DBH measurements have become a common practice. While variability in repeat measurements of DBH can occur due to placement of the measuring tape and tree morphology [50], citizen or community scientists can easily measure and electronically record DBH with low-cost equipment coupled to smartphones, thereby enabling a census of large numbers of trees. Such measurements can help to obtain an accurate snapshot of the urban forest [51], and in smaller studies, community science data has also been used for determining growth [52]. Community scientists expand the scope of the work feasible for ecological studies [53] while also elevating public interest and understanding [54, 55].

In addition to empirical field estimates, growth predictions can be made from existing databases, such as iTree and Urban Tree, based on trees from representative cities [52, 56, 57]. The models can then be adjusted for local context, including the number of frost-free days, location of tree (e.g., street tree, park, or forest), crown light exposure, health of the tree, and size of the tree [e.g. 58]. These generalized, broad-based models are based on limited tree data from select cities, but are regularly used to make predictions at both the tree level and regional level.

The New York City Parks and Recreation Department has supported a decadal street tree census since 1995, yielding data for 1995, 2005 and 2015. These data are available via the NYC OpenData portal (https://opendata.cityofnewyork.us/). Most recently in the 2015 NYC TreesCount! Tree census, 666,134 street trees were mapped, including characteristics of species, DBH, and level of stewardship, by over 2200 community scientists, TreeCounts paid interns, and NYC Parks Department employees [1]. While the data within these censuses could provide unprecedented insight into street tree growth rates across NYC, inaccurate geolocation of the surveyed trees in the 1995 and 2005 datasets has prevented such analyses.

Drawing on two iterations of the NYC Tree Census (2005 and 2015) collected by both paid staff and community scientists, we established a non-invasive method for determining urban street tree growth in NYC that did not rely on tree geolocation data. First, we applied a novel approach to align the NYC tree census data from 2005 and 2015 based on address and tree species. Next, we conducted two ground truthing surveys to verify the approach. Third, we calculated individual tree growth of 126,362 street trees in NYC representing 59 species. Next, we analyzed how the biological factors and urban form impact the rates of tree growth across NYC. Finally, to better understand the environmental justice implications, we explored how the distribution and variations in tree growth might impact local communities of varying socio-demographic backgrounds. Understanding the factors influencing urban tree growth will help urban planners make better decisions around green infrastructure that maximize ecosystem services for all communities across urban areas for decades into the future.

## Methods

### New york city tree census data

This study uses the 2005 and 2015 NYC tree census data to calculate growth rates. We used the 2005–2006 TreesCount! Street Tree Census (termed 2005 data) [59] and the 2015–2016 TreesCount! Street Tree Census (termed 2015 data) [1] that are available from the NYC Open Data Portal (https://opendata.cityofnewyork.us/). The 2005 census surveyed 592,130 trees and the 2015 census surveyed 666,134 trees. The surveys were conducted by both community scientists and paid employees. In 2005, the dataset does not indicate who collected the data. In 2015, the dataset indicates if the data were collected by community scientist volunteers, TreesCount staff who were hired specifically to help with the tree count, or NYC Parks Department employees. The dataset contains information pertaining to location (e.g., address, latitude, and longitude), tree species, tree diameter at breast height (DBH), descriptions of the tree health, and descriptions of the tree environment such as the tree pit and sidewalk. Errors in the latitude and longitude from the 2005 dataset make it challenging to use this data to definitively locate the same tree in the 2005 and 2015 data. The methodologies to determine latitude and longitude were improved in 2015 by using block edges and distances from intersections to properly locate trees. Because of improved location data, detailed street tree maps are available (https://tree-map.nycgovparks.org/). In future tree censuses, it will be possible to relocate each tree, but this is currently not possible. To align the 2005 and 2015 data and calculate growth rates, this study tests and verifies an address-based, tree-species approach.

**Fig 1:**
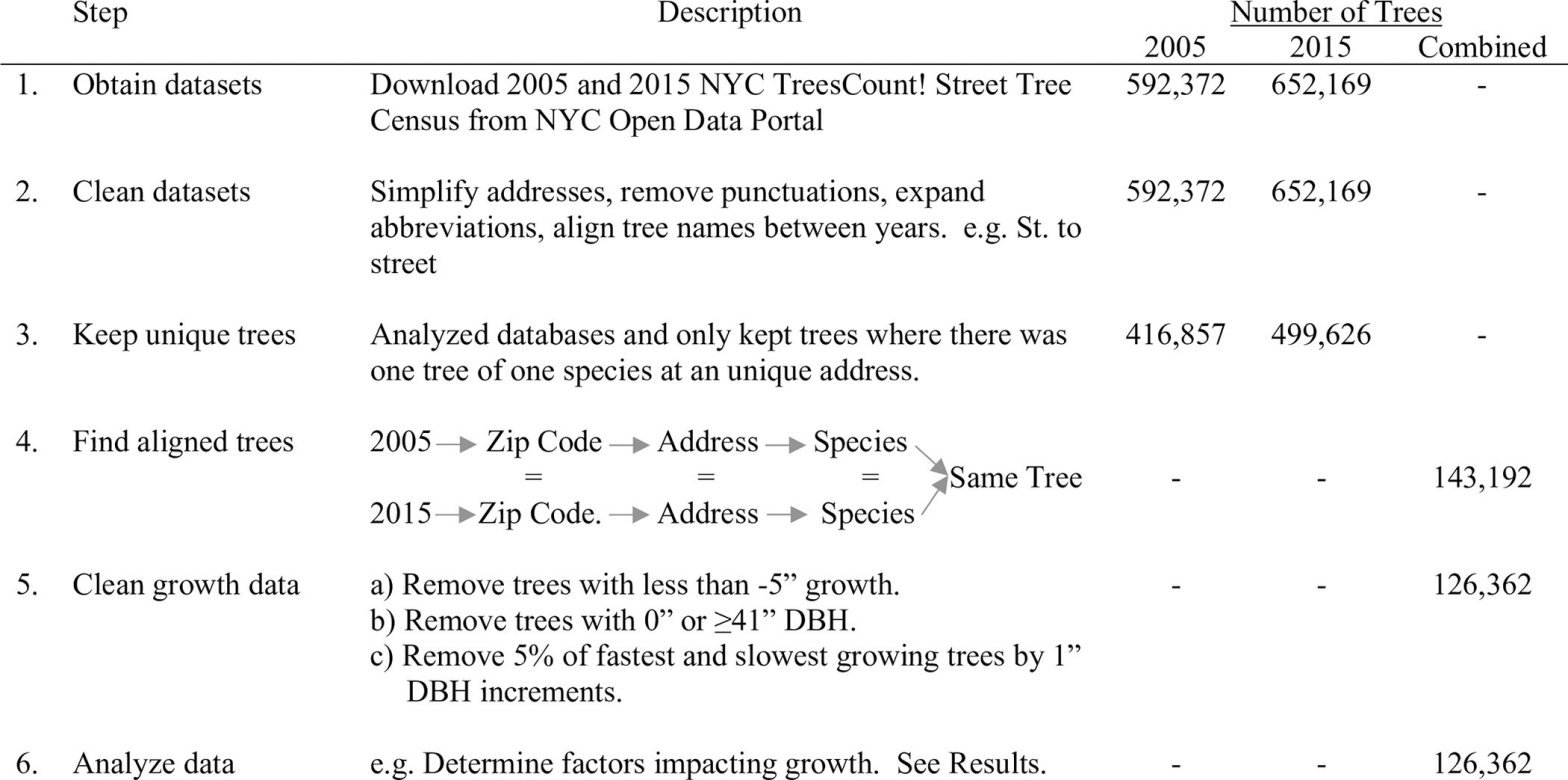
Summary of data processing, cleaning and analysis methods for the 2005 and 2015 datasets. The number of trees used at each step is shown for before and after alignment.

The downloaded tree census data were cleaned to enable alignment of the datasets (Fig 1, steps 1 and 2). A minimalistic approach was taken. The common names of each tree were made uniform. The addresses were simplified to remove punctuation and expand abbreviations. Multiple trees of the same species at one address were removed to avoid overlap (Fig 1, step 3). In order to overcome the errors in latitude and longitude in locating the trees, a methodology was developed where the datasets were aligned by ZIP Code, address, and tree species (Fig 1, step 4). Both ZIP Code and address were used as street names and building numbers can repeat across NYC. This resulted in alignment of trees between datasets, including unique trees in both data sets that were the same species at the same address and ZIP Code with no redundancy. For example, if the combined 2005 and 2015 datasets showed one Gingko and one Red Oak at the same address and ZIP Code, they would both align and be utilized. However, if one address had two London Planetrees, they both have to be removed for non-uniqueness. This alignment method could introduce potential bias if the same species is planted in series throughout a neighborhood and thus might be removed from the database more frequently in particular areas of the city. It is also possible that areas with few addresses, such as larger commercial or industrial buildings or along parks, may have more trees listed per the fewer addresses and thus also removed.

After database alignment, the tree growth data required further cleaning through a multistep method based on descriptive statistics of the measured DBH values (Fig 1 Step 5). Descriptive statistics of the cleaned dataset are presented in the results section. The database cleaning was designed to remove three potential errors (Fig 1, step 5): negative growth rates (e.g., a tree was removed and replaced with a smaller sapling of the same species between 2005 and 2015), large errors or outliers in DBH measurements, and the erroneous alignment of trees.

Some trees (n = 18,238, 12.7%) in the dataset showed negative growth. Three potential mechanisms of negative growth were investigated and used to determine if some or all of the trees should be removed from the dataset. First, clear errors were observed; for example, some 2005 data appear to be recorded as circumference and not diameter. Second, ground-truthing indicated that if a mature tree was replaced with a sapling of the same species, this would be recorded as a large negative tree growth event and these should be removed. Third, rounding or variability during measurement could cause errors in tree growth calculations. For example, if a tree didn’t grow, a slight error in measurements that resulted in rounding up or down in the 2005 and 2015 census and could easily lead to apparent growth of plus or minus two inches. Thus, we chose a conservative approach that growth of -5 inches was possible and part of measuring errors and used this as a cutoff (Fig 1, step 5a). The observed distribution of growth rates supports this choice.

To finish cleaning the data, we removed further outliers of trees that appeared to grow ‘too fast’ or ‘too slow’. First, small saplings with a 2005 DBH equal to 0” (n = 52) or trees with 2005 DBH 41” and larger (n = 1266) in the 2005 data were removed (Fig 1 step 5b). This step removed trees with DBH that had been rounded down to 0” and the few large trees (<1%) that might skew data. In order to remove further outliers, trees were grouped by their 2005 DBH in 1” increments to account for smaller trees growing at faster rates. We removed 5% of trees with the smallest and largest growth in each 1” increment (Fig 1 step 5c). The final aligned NYC Tree Growth dataset included 126,362 trees (21% of 2005 data and 19% of 2015 data based on initial counts; and 88% of initially aligned trees (see results)). Even with the removed trees, this newly created NYC Tree Growth dataset appears to be the largest dataset available for tree growth and offers a unique opportunity to examine a multitude of factors that might impact tree growth across an urban landscape.

### Database validation

Two tree monitoring programs were undertaken to ground truth and verify the methods to align and clean the 2005 and 2015 datasets. First, in order to validate the database alignment methods, in Spring 2019, we ground-truthed locations with tree records in both the 2005 and 2015 datasets in the 10024, 10025, and 10027 ZIP Codes (hereafter NYC ground-truth validation). Once located, if a tree was present or accessible it was identified and the DBH measured to validate growth rates by comparing the 2019 data to 2005 and 2015 NYC data. If a tree was not present or accessible, the reason was noted to determine the likelihood of finding the same tree in the aligned database. Second, in order to further validate variations in tree growth rates observed in the decadal citywide database (e.g., by species and by initial tree DBH), we monitored the DBH of trees in a four-block zone in the 10027 ZIP Code approximately annually beginning in 2015 or 2016 through 2021 (hereafter NYC temporal validation). All DBH measurements were performed at a height of 4.5 feet and measured to the hundredths of an inch (NYC Tree census data was rounded to the whole inch).

### Statistical analyses

Variation in tree growth rates in the newly created NYC Tree Growth dataset were explored based on properties of the tree, stewardship, and the surrounding local environment (Table 1). Analyses accounted for biological properties, such as species, tree health, and DBH in 2005 as a proxy for tree age, stewardship of the tree, local urban form based on location, properties of the tree pit, street width, nearby land use zoning, building height, and population density (Table 1). The data utilized comes from the 2015 Tree Census, MapPLUTO, NYC Street Centerline (CSCL), the American Community Survey (ACS), and the CDC/ATSDR Social Vulnerability Index (SVI) (Table 1) [60–63].

**Table 1:** Parameters used in this paper to better understand the controls on tree growth. The numbers next to each name indicate the source of the data.

Description	Name	Data Type	Values
Common name for the tree species	spc_common^1^	Categorical	Species Name
Diameter at breast height in 2005	dbh_05^1^	Continuous	1–40 inches
Category of user who collected tree data	user_type ^1^	Categorical	Volunteer, TreesCount Staff, NYC Parks Staff
User’s perception of tree health	Health^1^	Categorical	Good, fair, poor
Location of tree in relation to curb	Curb_loc^1^	Categorical	OnCurb, OffsetFromCurb
The number of signs of stewardship around the tree	steward^1^	Categorical	None, 1or2, 3or4, 4ormore
Presence and type of tree guard	Guards^1^	Categorical	Harmful, Helpful, None, Unsure
Sidewalk damage adjacent to the tree	sidewalk ^1^	Categorical	Damage, NoDamage
Indicates any problem with the tree roots	root_blocked^1^	Categorical	Yes, no
Indicates any problem with the tree trunk	trunk_altererd^1^	Categorical	Yes, no
Indicates any problem with the tree branches	Branch_problems^1^	Categorical	Yes, no
Name of borough where the tree is located	Borough^1^	Categorical	Manhattan, Bronx, Brooklyn Queens, Staten Island
The side/quadrant of the road where the tree is located	Roadside_location^2^	Categorical	North, South, East, West
Width of street	ST_WIDTH^3^	Continuous	Width of the street where the tree is located. Range:0–90 ft. Mean: 34.2 feet
Zoning of tax lot	LandUse^4^	Categorical	Landuse of the lot closest to the tree: Multifamily, open&recreation (open&ec), Single family, non-residential (non-res)
Built Floor Area Ratio. Indicates height of building.	BuiltFAR^4^	Continuous	Value of building closest to tree: Range:0–85, mean:1.2.
Population density of the census tract where the tree is located.	Pop_density^5^	Continuous	Value from the census tract where the tree is located. Range 0–481,072, mean 42732 persons per square mile
Index of the CDC/ATSDR Social Vulnerability Index (SVI) of the census tract where the tree is located	SVI_Vulnerability^6^	Continuous	Value from the census tract where the tree is located. Values are 0–1 with 1 being more vulnerable. Range: 0.02–1.00, mean: 0.54

1. Data obtained from the NYC Tree Database

2. Roadside location was calculated using the ArcGIS Pro software by determining the road direction by applying the linear directional mean function of the NYC street centerline data and then linking the trees to their closest road section using the Near function and finally assigning the roadside location to the trees.

3. Street widths were also obtained from the NYC Street Centerline data. They were attributed to the trees as the width of each tree’s respective closest road section, as described in the footnote 2.

4. Data obtained from MapPLUTO [62]. They were attributed to the trees using the Spatial Join function in ArcGIS Pro.

5. Data obtained from the American Community Survey (ACS) [63]. They were attributed to the trees using the Spatial Join function in ArcGIS Pro at the census tract scale.

6. Data obtained from CDC/ATSDR Social Vulnerability Index (SVI) [60, 64]. They were attributed to the trees using the Spatial Join function in ArcGIS Pro. Data were not used in the OLS regression model but for comparisons to residuals.

All statistical analyses were performed using Python v3.6.6 with Pandas v1.0.3 and Statsmodels v0.13 except the Generalized Variance Inflation Factor (GVIF) [65] to quantify collinearity which was calculated with the car package in R. All data and scripts (except ones done in ArcGIS) are available on GitHub (https://github.com/bmaillou/NYCTreeGrowth). In the final aligned decadal NYC Street Tree Growth dataset, growth rate was determined by calculating the difference in DBH (growth) between 2005 and 2015 for each individual tree and dividing by ten years. In the ground-truth and temporal validation datasets, growth rates were determined by calculating the Theil-Sen Slope as it minimizes the impact of outliers. The non-parametric Kendall rank correlation coefficient was utilized to assess the goodness of fit of the calculated growth rates. A student t-test was used when comparing two parameters and an ANOVA with the Tukey post-hoc test was utilized when comparing three or more parameters. An Ordinary Least Squares (OLS) regression was performed to determine the controls on growth rate. The goal of the OLS was to determine the effect of species, tree guards, and stewardship on observed growth rates along with the efficacy of using of community science volunteers. The OLS was controlled for both biological factors and urban form that could impact growth rates. This includes controlling for DBH in 2005 (“dbh_05”) since tree age is not known and younger trees generally grow faster, tree size was controlled for as a surrogate. The continuous data were normalized by subtracting the mean and dividing by twice the standard deviation to enable comparison with the categorical data [66]. The residuals or the difference between the predicted growth rates from the OLS and observed growth rates were used to determine areas where tree growth was faster or slower than expected.

## Results

### Growth rate observations

**Fig 2:**
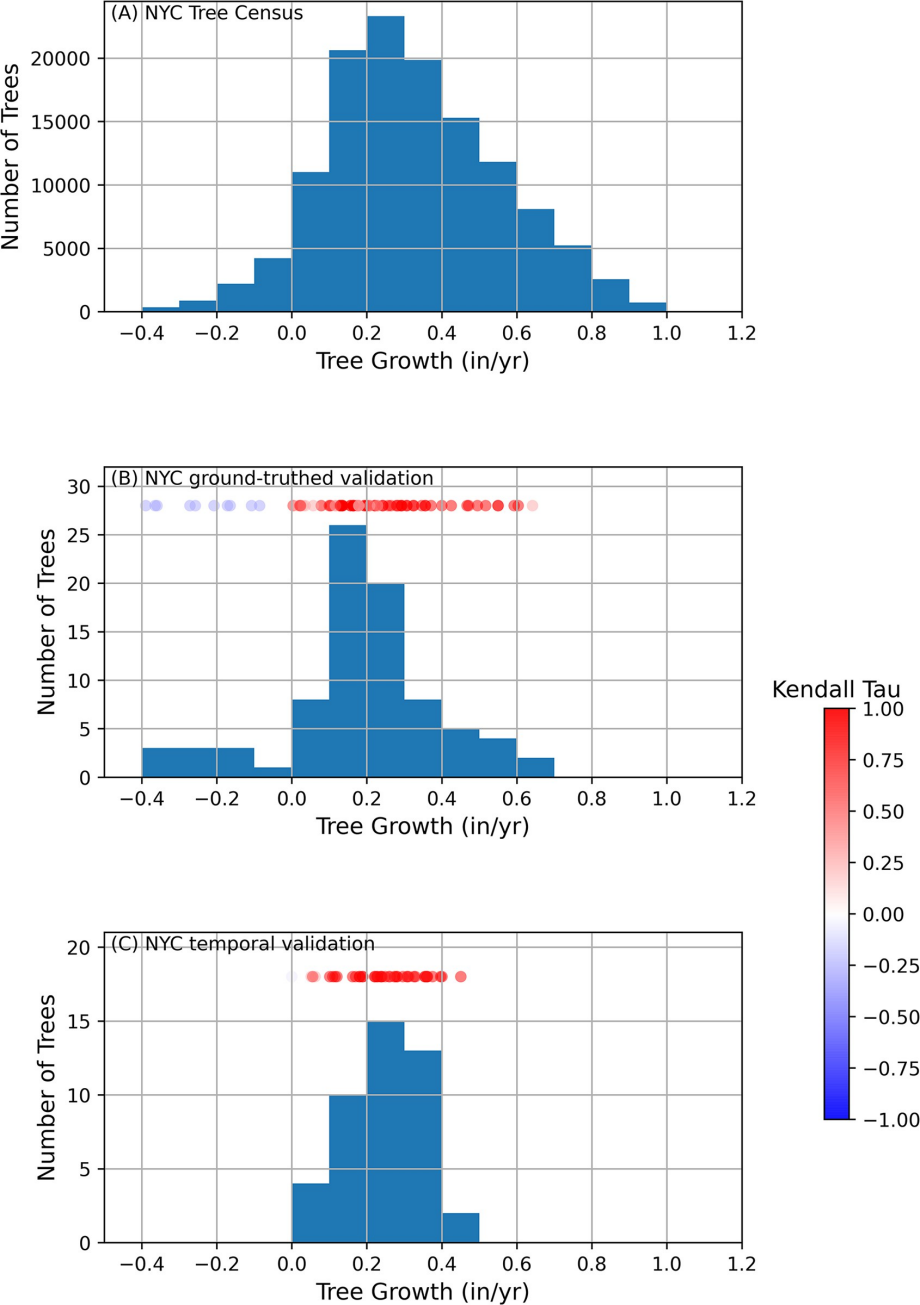
Histograms of tree growth from the different datasets used in this study. If a tree had three or more points the Kendall tau is included to show the goodness of fit for all trees. A +1 indicates a monotonic increase in DBH when growth rates are calculated and a -1 indicates a monotonic decrease. Bins of 0.1 in/yr were chosen as this is the smallest possible increment for the NYC Tree census data. (A) Tree growth in trees from the NYC Tree censuses. (B) NYC ground-truthed validation, growth rates of 86 locations with tree records in both the 2005 and 2015 datasets that were revisited and DBH measured. (C) NYC temporal validation, growth rates of 44 trees in a four-block zone in the 10027 ZIP Code measured approximately annually between 2015 and 2022.

Our alignment of the 2005 and 2015 New York City (NYC) Tree census databases by tree species, ZIP Code, and address (Fig 1) resulted in 143,192 trees with two DBH measurements. After further cleaning of the database (Fig 1), the final NYC Tree Growth dataset included 126,362 trees of 59 species. Of the 59 species, 14 species included more than 1000 individuals each and an additional 10 species included 100 to 1000 trees (Fig 2A, Table 2). The mean street tree growth rate was 0.275±0.233 in/yr (+/- 1 standard deviation; 0.699±0.594 cm/yr) with a minimum of -0.5 in/yr (-1.27 cm/yr) and a maximum of 1.6 in/yr (4.06 cm/yr). 7706 (6%) of trees had a negative growth rate. The remainder of the results are presented in inches as the NYC databases are in inches and the data are rounded to the nearest inch to minimize conversions and errant decimals. Inches are likely most relevant to the policy makers. 118,585 (94%) of trees had a growth rate between 0 and 1 inches/yr (Fig 2A).

**Table 2:** Summary of the newly created NYC tree growth dataset. The growth dataset grouped by species is compared to the top trees in the 2015–2016 TreesCount! Street Tree Census (termed 2015 data) as taken from NYC data [1]. The growth database is missing two trees that are in the top 15 of the 2015 data: Cherry (rank = 7) and Sophora (rank = 10).

	Growth Database				2015 Data
Common Name	Abundance Rank	Number of Trees	Mean DBH 2005 (in)	Mean Growth Rate (in/yr)	Abundance Rank
London Planetree (*Platanus x acerifolia*)	1	32,058	22.5	0.163	1
Honeylocust (*Gleditsia thriacanthos*)	2	15,974	9.1	0.356	2
Callery Pear (*Pyrus calleryana*)	3	15,903	6.6	0.334	3
Norway Maple (*Acer platanoides*)	4	13,149	13.4	0.174	5
Pin Oak (*Quercus palustris*)	5	11,563	16.8	0.352	4
Littleleaf Linden (*Tilia cordata*)	6	6,343	9.7	0.318	6
Ginkgo (*Ginkgo biloba*)	7	5,518	9.0	0.257	9
Green Ash (*Fraxinus pennsylvanica*)	8	4,893	10.9	0.366	12
Red Maple (*Acer rubrum*)	9	4,116	10.1	0.273	11
Silver Maple (*Acer saccharinum*)	10	3,948	20.4	0.265	14
Japanese Zelkova (*Zelkova serrata*)	11	3,643	8.2	0.425	8
Sweetgum (*Liquidambar styraciflua*)	12	2,620	11.0	0.308	15
Silver Linden (*Tilia tomentosa*)	13	1,149	6.7	0.510	17
Northern Red Oak (*Quercus rubra*)	14	1,026	12.7	0.375	16
American Linden (*Tilia americana*)	15	868	10.8	0.399	13

**Fig 3:**
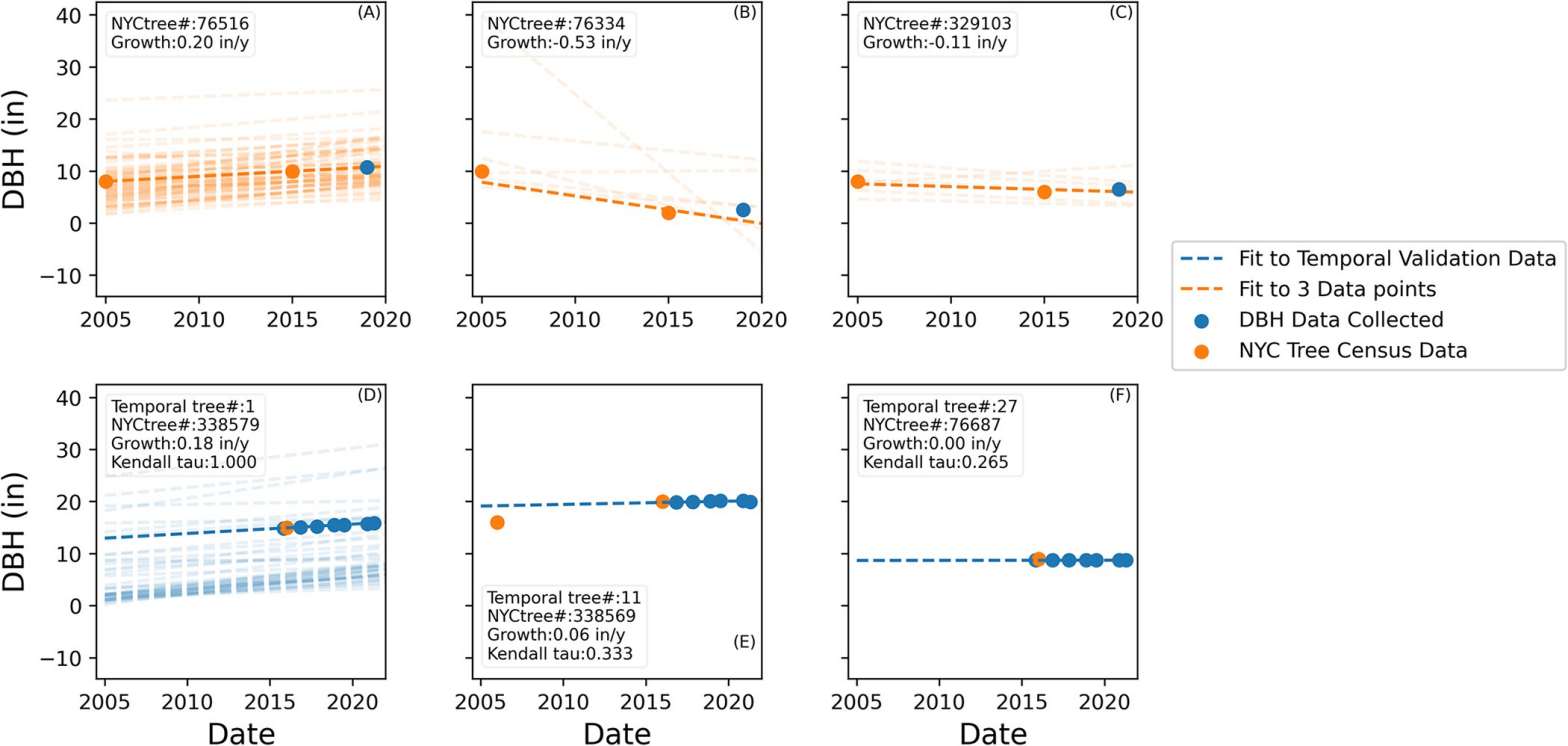
Diameter at breast height (DBH) versus time for select trees from the different studies to highlight the variety of responses observed. Orange points are from the NYC Tree censuses and blue points were measured in ground truthing and temporal validation. The color of the best fit lines represents the points they were fit to. A-C) represent trees that were remeasured in ground truthing. D-F) represent trees that were measured multiple times in temporal validation of growth rates. The points and darker line represent a typical example tree and the lighter lines show the remainder of the data for each type. (A) Trees with positive growth between the 2005 and 2015 NYC datasets; representative of the majority of cases. (B) Trees with a DBH decrease of less than -5 in between the 2005 and 2015 NYC datasets. (C) Trees with a DBH decrease of between 0 and -5 in for 2005 and 2015 NYC datasets. (D) A typical tree that was monitored annually and has linear growth along with all other samples. (E) The only tree for which we have two NYC Tree census points and annual data points. It is not clear why only one tree had 2005 data. (F) A tree that was measured annually but did not grow highlighting that zero growth is possible.

To verify that the same trees could be re-located and the database alignment and cleaning methods were justified, ninety-one locations in the cleaned NYC Tree growth dataset were visited and ground-truthed. Eighty-six trees were located; two trees were missing, two were blocked by construction, and one was in the process of being cut down. Out of the eighty-six trees, the majority (n = 73, 85%) had positive growth rates from 0.0 to 0.64 inches/yr, consistent with NYC Tree growth dataset (Figs 2B and 3A–3C). Fifty-three trees had a Kendall tau equal to one indicating the three DBH measurements (2005, 2015, 2019) monotonically increased (e.g., Fig 3A). Seven trees (8%) had a DBH that decreased by 5 inches or more between 2005 and 2015 and upon inspection of the trees, pictures of the trees, and examining the data; five had obviously been removed between 2005 and 2015 and replaced with a smaller tree of the same species that then grew between 2015 and 2019 (e.g., Fig 3B), one appears to be a data entry error in the 2015 NYC data (1 instead of 10), and one appears to a measurement error in the 2005 NYC data. The tree with the data entry error still had a positive slope. Based on these findings of the ground-truth validation, we justified removing trees from the database in which a DBH decreased by 5 inches or more. Eight trees had more ambiguous negative growth based on the 2005 and 2015 DBH values where measurement errors and rounding could possibly lead to a negative growth (Fig 2C) but only seven of these when including the 2019 data were represented by a negative slope/Kendall tau (Fig 2B).

To better understand growth, forty-four trees were monitored approximately annually starting in 2015 or 2016 until 2021 as a temporal validation of growth rates in urban NYC trees (Fig 3D–3F). All (100%) of the trees had a positive growth rate from 0 to 0.73 in/yr (Fig 3D–3F, Fig 1C). Twenty-eight (64%) of the trees had a Kendall tau of 1 (Fig 3D, Fig 2C). Thirty-six of the trees measured annually also had a datapoint from the 2015 NYC Tree Census Data with only one also having a datapoint from the 2005 Tree Census Data (Fig 3E). NYC Tree 338569 (Fig 3E) was linear when including the 2015 NYC data and the temporal validation data but not when including the 2005 NYC data. This could be due to a change in growth rate or a measurement error. Overall, our temporal validation highlighted annual measurements performed as part of this study were similar to data from the NYC Tree growth dataset.

### Spatial patterns of tree growth, density, and size

**Fig 4:**
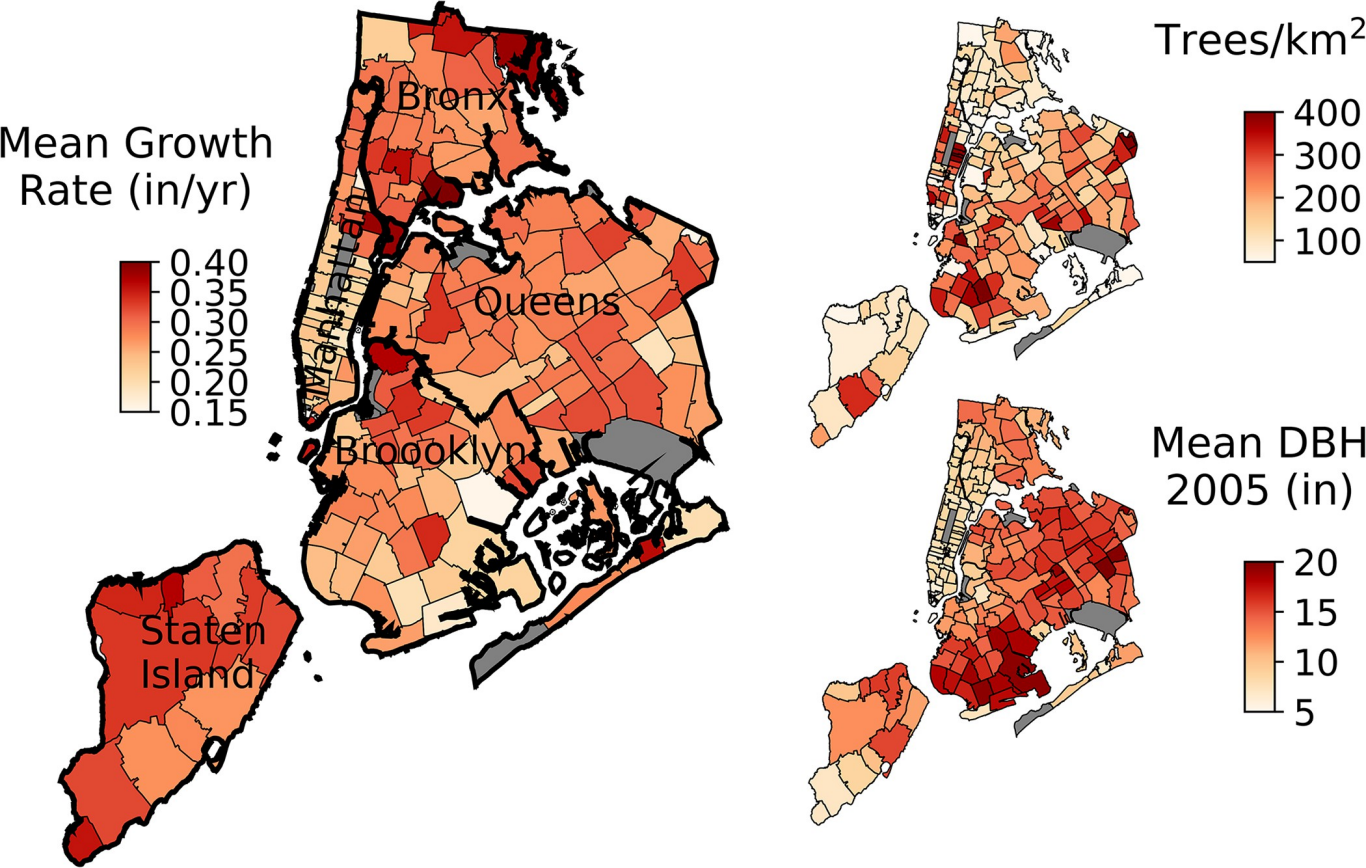
Maps of NYC tree data at the ZIP Code level. The large map shows mean growth rates whereas the two insets show the number of trees per km^2^ and the mean diameter at breast height (DBH). The data is only for the trees in the NYC Tree Growth dataset. The five boroughs of New York City are labeled and outlined in thicker black for reference for subsequent maps. The shapefiles are available from the NYC Open Data Portal (https://opendata.cityofnewyork.us/).

The street tree growth rates, density, and size varied across the city when averaged by ZIP Code (Fig 4). Mean growth rates were the slowest in Manhattan and southern Brooklyn and faster in Staten Island along with parts of Queens and the Bronx (Fig 4, boroughs are labeled on the map). Public street tree density was greatest in much of Manhattan, southern Brooklyn, and eastern Queens. Street trees also varied in size across the city (Fig 4) where Manhattan and the southern Bronx had the smallest DBH trees and southern Brooklyn and eastern Queens had trees with larger DBH.

### Factors impacting tree growth

**Fig 5:**
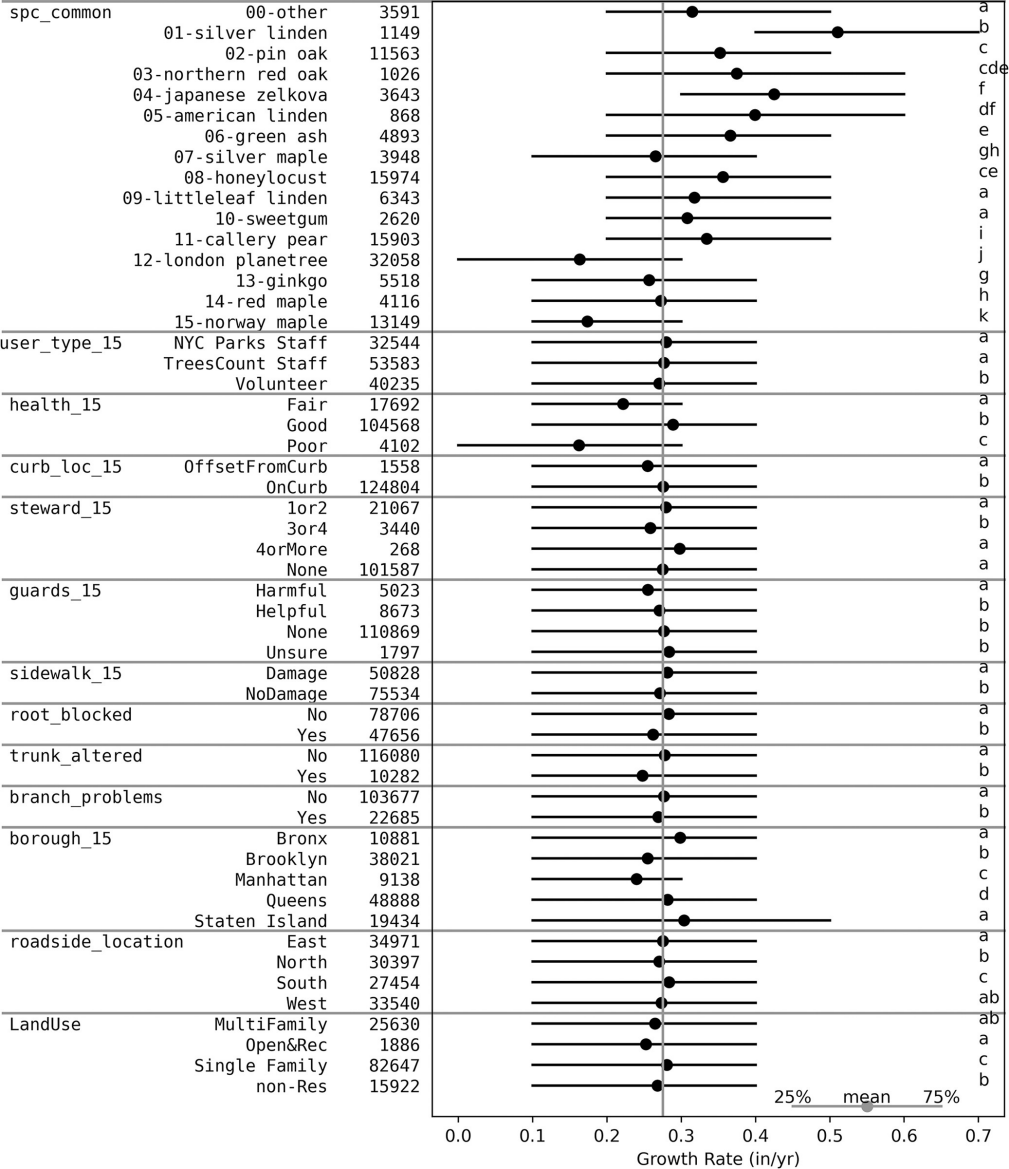
Forest plot to summarize impact of categorical data (Table 1) on growth rates. The vertical gray line is the overall mean of 0.275 in/yr. Each horizontal line goes from the 25^th^ to 75^th^ percentile with the point being the mean. The letters on the right are the statistically significant groups as determined with an ANOVA and a Tukey post-hoc test within that subgroup if there are three or more parameters or a t-test when two parameters. Significant differences are determined based on p<0.05.

The data were further analyzed to better understand the parameters that impact tree growth and the observed spatial patterns. Initial analyses focused on how growth rates vary based on properties of the tree (e.g., species, health, presence of root damage, etc), properties around the tree (presence of guard, location relative to curb, etc), and the surrounding urban form (street width, building height, etc) (Table 1). The largest variations in growth rate were observed by species (Fig 5, Table 2). Out of the 15 most abundant species, Silver Linden has the fastest observed mean growth rates (0.510±0.213 in/yr for 1,149 trees). London Planetree is the most abundant tree (n = 32,058) but has the slowest mean growth rate (0.163±0.215 in/yr).

The mean tree growth rates were compared by observed health, stewardship, and surrounding environment of the tree (Fig 5). Trees categorized as “Good” health in 2015 (health_15) grew faster (0.289 in/yr) than trees categorized as “Fair” (0.222 in/yr) and “Poor” (0.162 in/yr; Fig 5). Trees located (curb_loc_15) “OnCurb” (0.276 in/yr) grew faster than “OffsetFromCurb” (0.255 in/yr). Signs of Stewardship in 2015 (steward_15) did not improve mean growth rates; trees classified with “3or4” signs of stewardship had the slowest (0.258 in/yr) growth rate compared to more stewardship (“4orMore”) (0.298 in/yr) or less stewardship (“None” (0.275 in/yr) or “1or2” (0.279 in/yr)). Trees with “Harmful” guards in 2015 (guards_15) such as tall and narrow or guards that allow soil to build up against the trunk [67] had slower mean growth rates (0.255 in/yr) than “Helpful” (0.271 in/yr), “None” (0.276 in/yr) or “Unsure” (0.283 in/yr). Trees located by a damaged (“Damage”) sidewalk in 2015 (Sidewalk_15) had faster mean growth rates (0.281 in/yr) than those trees located by sidewalks with “NoDamage” (0.271 in/yr). Having a root blocked (“root_blocked” “yes”), trunk altered (“trunk_altered” “yes”) and branch problems (“branch_problems” “yes”) were all associated with slower mean growth rates than compared to each “no” (Fig 5). The borough the tree was located in (borough_15) had a significant impact on growth. The Bronx (0.299 in/yr) and Staten Island (0.304 in/yr) have the fastest mean growth rates whereas Manhattan (0.240 in/yr) has the slowest. Trees located on the “South” side of the street (roadside_location) grew the fastest. Trees in front of “Single Family” in LandUse grew faster (0.281 in/yr) than “non-Res” (non-Residential) (0.268 in/yr), “MultiFamily” (0.265 in/yr) and “Open&Rec” (0.252 in/yr).

Using Pearson correlation coefficient, we examined the correlation between continuous variables on the observed growth rates. Growth rates were negatively correlated with the DBH in 2005 (“tree_dbh_05”), Built Floor Area Ratio (BuiltFAR; higher number the taller the building), population density (“pop_density”), and street width (“st_width”) (Table 3). Except for the r for “tree_dbh_05” (r = -0.373) the other r values were much smaller but still significant (Table 3).

**Table 3:** Pearson correlation coefficients. The data is between growth rates from the NYC growth database and continuous data (Table 1) that may impact growth rates.

Parameter	Correlation coefficient	p-value
DBH in 2005 (tree_dbh_05)	-0.373	<0.001
Built Floor Area Ratio (BuiltFAR)	-0.046	<0.001
population density (pop_density)	-0.038	<0.001
street width (st_width)	-0.011	<0.001

### Community science data

**Fig 6:**
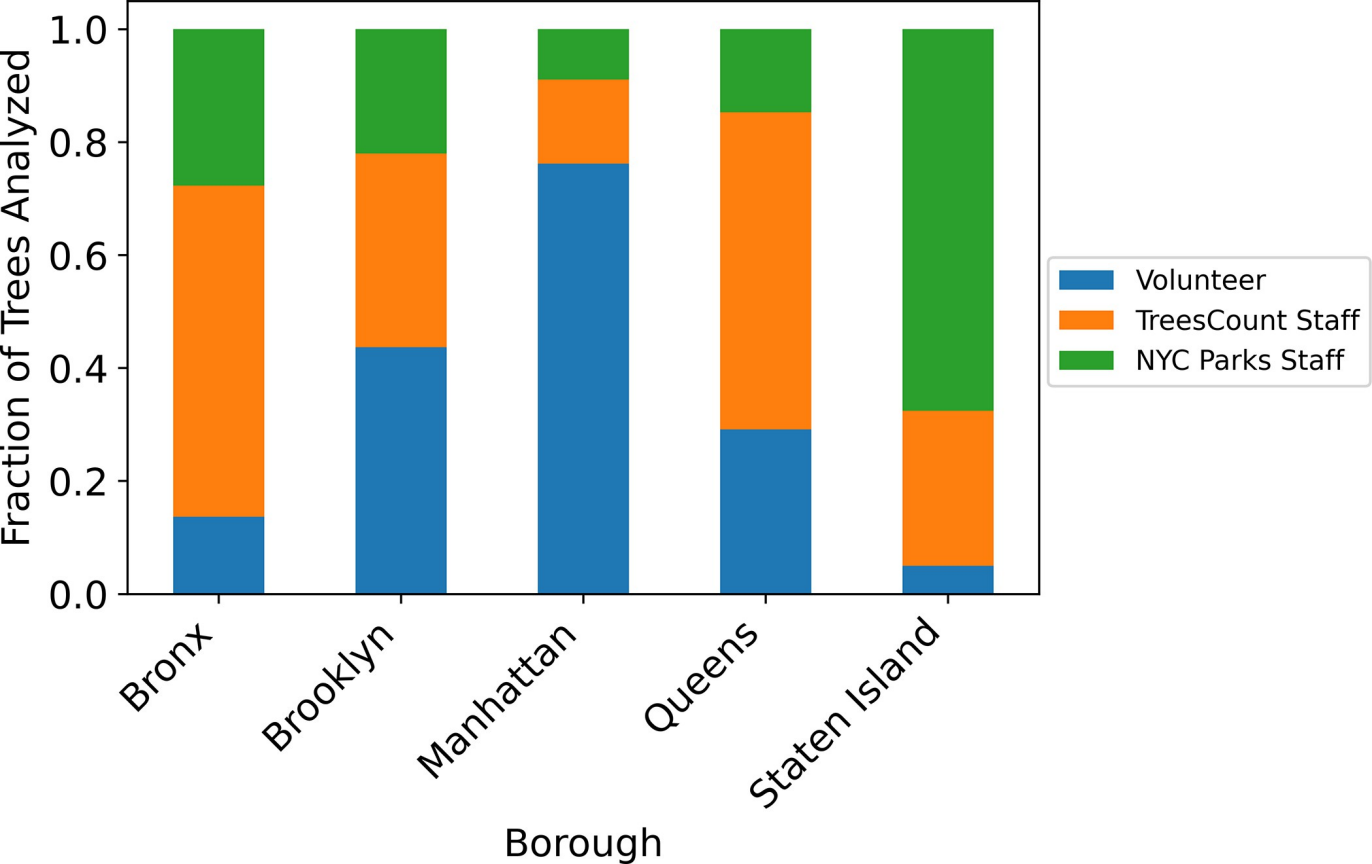
Percentage of trees measured by volunteers, TreesCount paid staff and NYC parks staff for each borough.

The 2015 NYC Tree census was conducted by three different groups; NYC Parks staff, TreesCount paid staff, and volunteers. Only the 2015 dataset denotes who made the measurement; the sources of measurement data is not available for the 2005 data. In some studies, experts replicate a fraction of measurements by community scientists to check for accuracy. In the 2015 data, trees were only analyzed once by one group and thus direct comparisons between experts and community scientists are not possible but with the large dataset it is possible to compare the overall values. The NYC Parks staff measured 26% of the trees. Mean growth rates of trees measured by NYC Parks staff (0.279 in/yr) were not significantly different than trees measured by TreesCount staff (0.277 in/yr), which measured 42% of the trees (Fig 5). Volunteers measured 32% of the trees and had a significantly lower mean growth rate of 0.270 in/yr (Fig 5). However, volunteer measurements were spatially heterogeneous. Volunteers measured 76% of the trees in Manhattan which contrasts to only 5% of the trees in Staten Island measured by volunteers (Fig 6). The spatial heterogeneity indicates that volunteers and staff most likely measured different species of trees of different sizes in different boroughs with different growth rates.

### Ordinary least squares regression analysis

**Fig 7:**
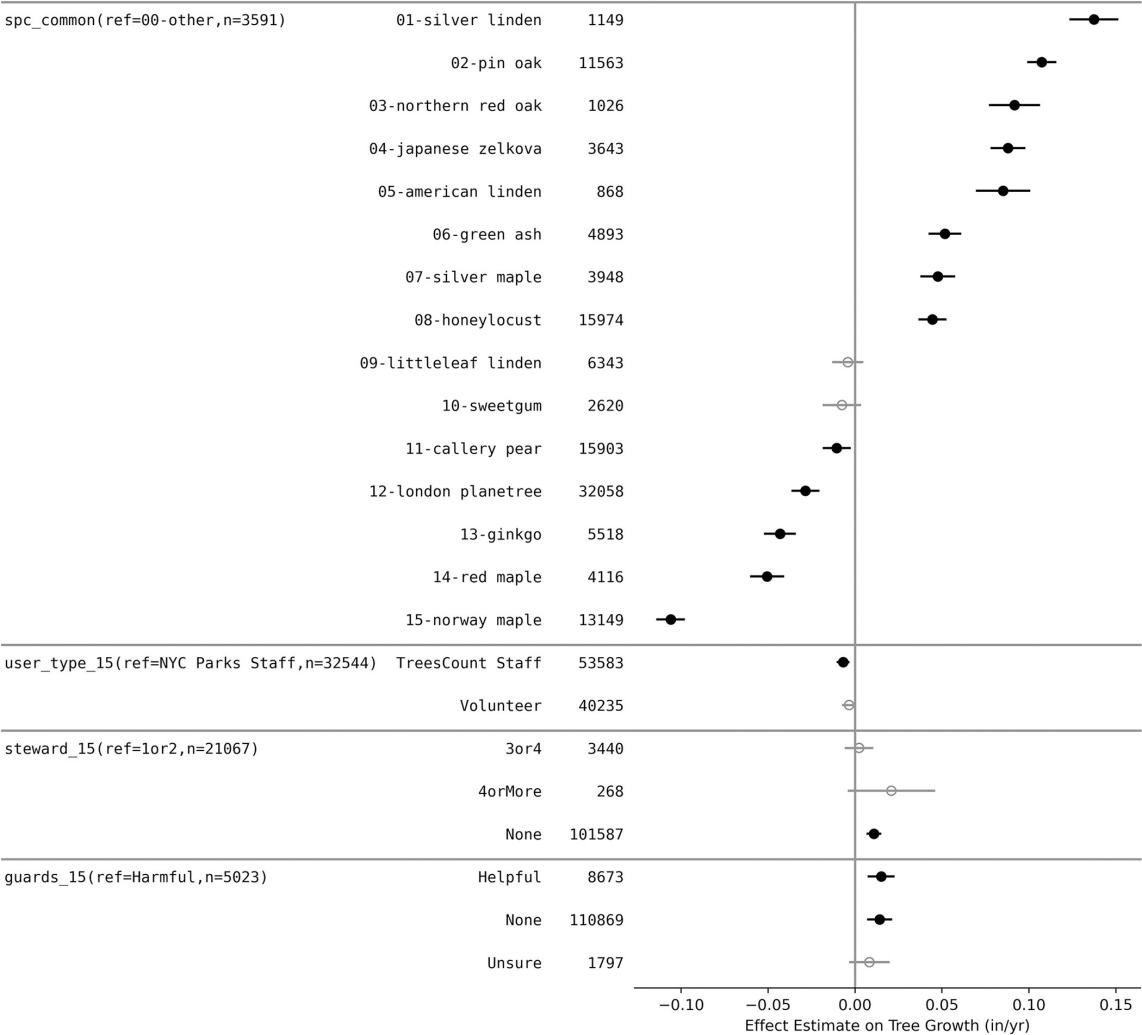
Ordinary least squares regression of factors controlling growth rate data. The parameters are shown by Effect Estimate on Tree Growth (in/yr). If the Effect Estimate intersects zero, it is not significant and grayed; if the Effect Estimate is significant at the 0.05 level, it is shown in Black. Analysis includes the 15 most common species in the growth database (shown here and Table 2). All other species were grouped into ‘other’ and used as the reference. The adjusted R^2^ was 0.252 with a root mean square error (RMSE) of 0.202 in/yr.

Given the large number of factors that had significant variations with tree growth (Fig 5), a multiple linear regression was performed (Fig 7) in order to explore the relative importance of factors on tree growth. All factors from Fig 5 were included or controlled for in the model. The results were controlled for biological factors (properties of the tree) and the local urban form (properties of the area around the tree, and nearby built environment, Table 1, Fig 5). This includes controlling for tree size (dbh_05) as a surrogate for age since younger trees generally grow faster and negatively correlates with growth rates (Table 3). Two of the parameters that were controlled for (Roadside_location and trunk_altered) were not significant, but were still left in the analysis since they were significant in Fig 5. The Generalized Variance Inflation Factors (GVIFs) were all less than three indicating minimal collinearity.

Overall, tree species had the biggest effect on growth rates. Silver Linden and Pin Oaks had largest positive effect on growth rates whereas Norway Maples, Red Maples, and Gingkos had the smallest negative. The effect between the slowest and fastest species was 0.243 in/yr or 2.43 inches in DBH growth between the decadal surveys (Fig 7). Differences in stewardship had only a small effect on observed growth rates. Increasing signs of stewardship (steward_15) were associated with no effect on growth rates and only trees with no sign of stewardship had a small positive effect of 0.011 in/yr. Again, the presence of tree guards (guards_15) had mixed results. Having no guards (“None”) (0.014 in/yr) or “Helpful” (0.015 in/yr) both had a positive effect of increasing growth rates as compared to “Harmful” guards. There were statistical differences on growth rates based on who measured the trees; TreesCount Staff had a small (-0.005 in/yr) but significant negative impact on growth rates whereas volunteers were no different than New York City Park Staff.

### Comparing growth to social vulnerability

**Fig 8:**
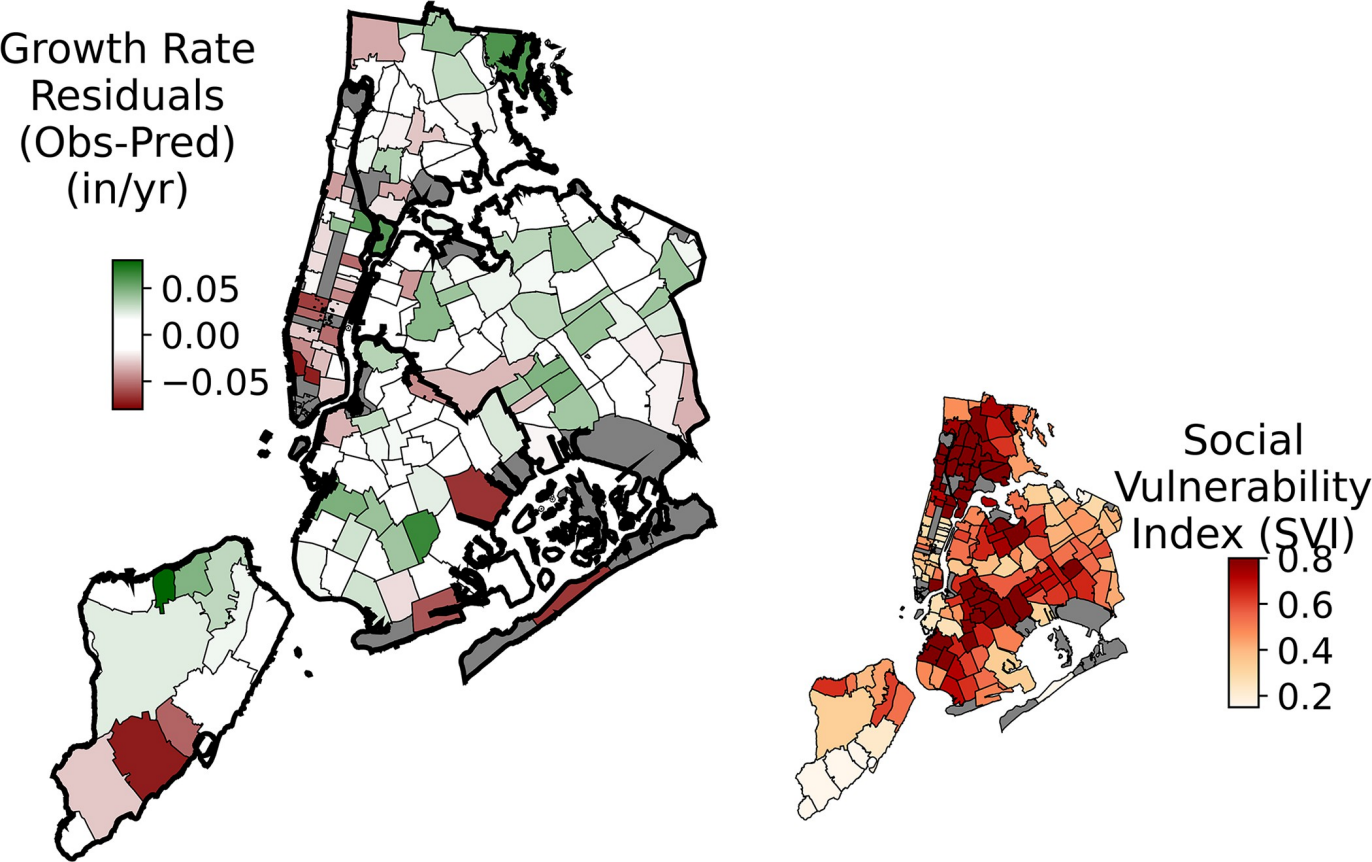
Residuals between observed and predicted growth rates averaged by ZIP code. The Predicted growth rates were based on ordinary least squares (OLS) regression excluding borough. Positive residual values indicate faster than expected growth rates and negative values indicate slower than expected growth rates. The inset shows the CDC/ATSDR Social Vulnerability Index (SVI) with higher numbers indicating more vulnerable neighborhoods. The 19 ZIP codes with less than 100 trees and ZIP codes 11224 and 11691 which were heavily flooded during Hurricane Sandy were removed leaving a total of 155 ZIP Codes. The spatial data are publicly available from the NYC Open Data Portal (https://opendata.cityofnewyork.us/ and Center for Disease Control Agency for Toxic Substances and Disease Registry Social Vulnerability Index (http://svi.cdc.gov).

**Fig 9:**
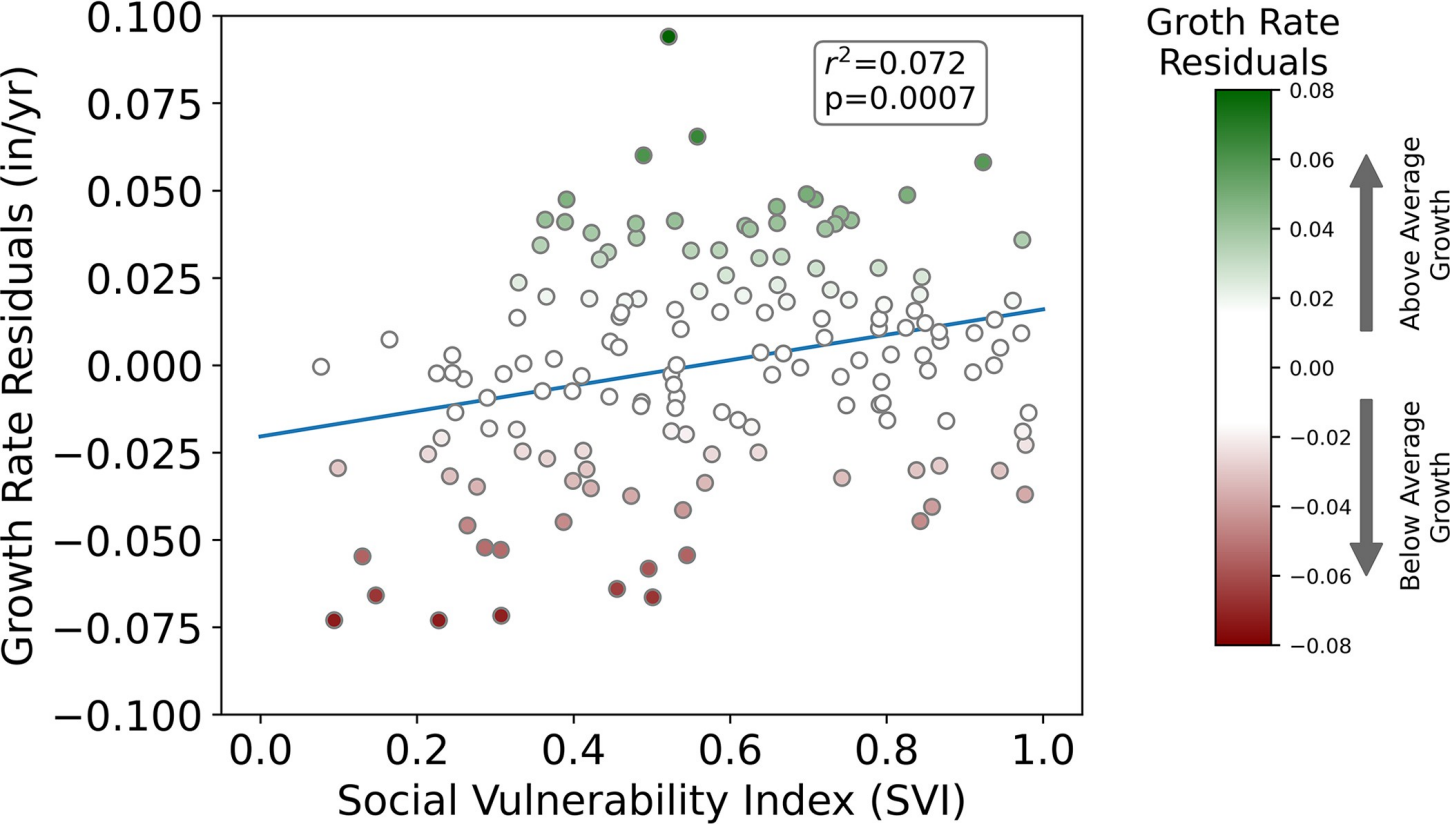
The CDC/ATSDR social vulnerability index (SVI) versus growth rate residuals. The symbols are colored by growth rate residuals to match the y-axis and the colors on the main map from Fig 8.

To determine if the growth of street trees could be a mechanism to help address past injustices and unequal tree distribution, we examined if growth rates are faster or slower than expected in neighborhoods of high social vulnerability across NYC. Overall, our results indicate that street trees are growing at faster than expected rates in areas of high social vulnerability. The OLS model of tree growth was utilized to determine if tree growth was under- (growing slower) or over-performing (growing faster) compared to predictions with growth rates averaged at the ZIP Code level (Fig 8). In this comparison the ordinary least squares regression was performed again but without controlling for borough (borough_15) which was used in the previous model. This change was made in order to remove this arbitrary spatial correction and resulted in the Roadside_location (a controlled variable) becoming significant with a small effect. Middle and southern Manhattan, southern Brooklyn and Southern Staten Island all had negative residuals and slower than expected growth rates whereas large portions of Brooklyn, Queens, and Staten Island have higher than expected growth rates (Fig 8). The most vulnerable populations based on the CDC/ATSDR Social Vulnerability Index (SVI) of the census tract where the tree is located 1) are centered in Northern Manhattan, the Bronx, northern Queens and central Brooklyn (Fig 8). Across New York City, we found a significant positive relationship between SVI and growth rate, such that in areas of higher SVI, growth rates are faster than predicted (i.e., the residuals increase; Fig 9).

## Discussion

Every 10 years New York City (NYC) undertakes the monumental task of inventorying all street trees. This data has been used to understand ecosystem services in NYC and to plan more equitable future tree planting to improve the urban landscape [9]. However, from the time of planting it can take decades before trees reach maturity and their full benefits are realized; reliable estimates of growth rates could be used to help improve planning and design. To our knowledge street tree growth rates across the whole of NYC have not been previously published due to the challenge of aligning individual tree locations (latitudes and longitudes) across multiple surveys. The database of street tree growth rates presented here includes 126,362 street trees of 59 species. Based on ground truthing and a temporal validation with annual measurements between 2015–2021, the large number of trees in the growth database appears to parallel the 2015 Street tree census. Thus, while the database incorporates only 19% of 2015 street trees—given the methods used to exclude ambiguous individuals and ensure accurate growth estimates—the dataset appears to capture the overall pattern of NYC street trees and can be used to explore citywide patterns of growth and the factors influencing growth rates.

Across the city, the growth rates varied between -0.5in/year to 1.6in/year (mean growth rate = 0.275±0.233 in/yr). Our growth rates mirror those estimated in other studies from models such as iTree with growth rates between 0.2–0.3 in/year [27, 58]. However, in our database, of the 15 most abundant species in 2015, 10 species had growth rates over 0.3 in/year (Table 1). Species was the largest predictor of growth rates. For example, Silver Linden had the fastest average growth rate at 0.510 in/yr. On the other hand, London Planetree, the most abundant tree species in the growth database and the 2015 TreesCount! Street Tree Census, had the slowest growth rate at 0.163 in/yr. However, the London Planetree still had a negative effect on growth even when controlling for size which was largest mean DBH by species in 2005 suggesting these trees might grow slower than expected as typically London Planetrees are known to have fast growth rates [68]. Across species, growth rates were higher in trees without root blockage or problems reported with branches or trunk alteration. Street trees planted on the South side of streets had slightly, but significantly faster growth rates, which may reflect better sun exposure, compared to other street orientations but this effect disappeared in the full OLS model. Finally, trees located in single family residential zones had above average growth rates whereas those planted in open space had the slowest, which likely reflects older, slower growing trees located around park and open spaces. Based on the requirements of urban planners, tree species can now be chosen based on growth characteristics for each species, along with urban form characteristics, above already present generalizable models. However, further in-depth modeling is still needed to understand all the factors impacting growth rates in NYC.

Many previous studies have examined survival and mortality rates of street trees [86], but few examine growth rates. Estimates of turnover and mortality in urban trees varies widely depending on species, age, and size [69, 70]. One study in NYC found an annual street tree mortality rate of 4.4%, with higher more variable mortality rates for younger trees [71]. Assuming a 4.4% annual mortality rate (36% mortality over ten years given exponential decay), only 64% of the trees in the 2005 dataset would be present in 2015. The dataset of growth rates in this paper is significantly larger than other published datasets including the study from Montreal (~28,000 trees) [46] and databases used for growth estimates that contain about 20,000 trees [56, 57]. Assuming all trees are resampled (~600,000) at the next street tree census in 2025, the trees are explicitly located for realignment with the 2015 database, and a survival of 64% of trees over ten years, this growth dataset should further expand to approximately 380,000 trees or almost three times the current study. This would be an unprecedented database to support future street tree planting campaigns.

However, careful attention is needed for planning the next tree census. If the location techniques from the 2015 census are used it should be possible to relocate almost all the tree pits in 2025. An improved online map of the NYC Street Trees already makes it easy to find 2015 census data (https://tree-map.nycgovparks.org/tree-map). Furthermore, the data from the 2015 census can be used to predict and ground-truth the 2025 DBH measurements which should be measured more accurately than to the inch. Field scientists could be asked in real-time through the app to remeasure a tree if it falls outside predicted growth or to assess and document if something has changed. This should enable determinations of growth, mortality, removal, or replacement for all street tree locations and would be an impressive step forward for the NYC tree census. Finally, the 2025 census should seek to better capture parameters that may be impacting growth rates [44].

There are numerous other factors that can impact growth, including stewardship of street trees, soil properties, tree pit size and characteristics, and local idiosyncratic effects like construction and scaffolding around trees [3, 72]. Signs of stewardship and tree guards (short protective fences around tree pits) have been shown to decrease street tree mortality [71] however in this study the effects on growth are small and variable. The majority of trees had no sign of stewardship and this was associated with a positive effect on growth rates when compared to trees with stewardship. Furthermore, the absence or presence of a helpful tree guard had the same positive effect compared to those labeled in 2015 as a “harmful” tree guard. The variable effects of stewardship here might reflect the limited way in which stewardship was observed and recorded in the tree census or stewardship might be a secondary factor impacting growth. The NYC Parks Department is charged with maintaining the ~600,000 street trees, but with limited budget and capacity relies on local stewards, including residents, civic organizations, or building superintendents, to care for street trees. Other studies with more in-depth observational and ethnographic methods have shown the important impacts of stewardship for both the stewards and the trees [73]. Other tree properties than growth, such as mortality, along with other ecosystem services, such as connections to nature and feelings about the neighborhood, should guide the presence of guards and stewardship decisions.

Residents and community scientists are critical for undertaking and completing large scale monitoring projects. These projects would not be feasible without the help of volunteers; however, it is critical to evaluate and balance community science benefits with accuracy in data collection. In the 2015 NYC Tree Census dataset, volunteers who served as community scientists measured 32% of the trees. The raw data suggests that the volunteers’ measurements of DBH indicated slower estimates of growth rates, possibly due to measurements errors, compared to those taken by NYC staff (Fig 4). However, upon further investigation, it was found that the volunteers were concentrated in Manhattan, which is the borough with a high density of small, and slow growing, street trees (Figs 3 and 8). The ordinary least squares regression further supported the conclusion that the volunteer measurements were as reliable as paid NYC staff, as it showed no statistical difference in the observed effect between the volunteers and NYC Parks staff. Interestingly, the paid NYC TreesCount staff had a small but significant negative effect on growth rates indicating training methods might need to be revisited. This result is consistent with previous studies that shows volunteers routinely measure DBH to within an inch of experts, but may be less accurate in estimating other parameters [51]. With continued training of volunteers, the NYC Parks staff should be confident they are obtaining reliable DBH measurements.

The spatially explicit database highlights that even after controlling for biological factors and urban form that faster than expected growth rates are occurring in areas of with a higher Social Vulnerability Index (SVI) (Figs 7 and 8), and thus may serve to improve ecosystem service provisioning in those locations. Previous work, including in NYC, has clearly shown that inequalities exist in ecosystem services across urban environments with lower urban canopy coverage in low-income, communities of color [26, 74–78]. Street trees are one potential method for helping to combat this problem but require space for proper tree pits, planting, care, and time for growth. The Million Trees NYC planting campaign increased the number of trees in NYC [9], but did not fully address the stated equity goals or needs of underserved areas. Recent research highlights how the majority of trees were planted in existing open spaces, which are unequally distributed by race and class, and thus, historical disparities still persist across NYC [79, 80]. Given the historical unequal distribution of street trees [e.g. see 26, 81, 82, 83], the distribution of faster growing trees may be potential good news and a path forward for mitigating inequities within NYC. We found the areas of NYC with a higher Social Vulnerability Index have above average growth rates (Figs 7 & 8). Strategic plantings and optimal choices of tree species may help to lessen distributional inequalities over time. Along with urban planners, communities can utilize known growth rates across species to aid in making informed decisions that account for community-based needs to improve their green space [84, 85].
